# Digital phenotyping in molecular psychiatry—a missed opportunity?

**DOI:** 10.1038/s41380-022-01795-1

**Published:** 2022-09-28

**Authors:** Christian Montag, Daniel S. Quintana

**Affiliations:** 1 Department of Molecular Psychology, Institute of Psychology and Education, Ulm University, Ulm, Germany; 2 Department of Psychology, University of Oslo, Oslo, Norway; 3 NevSom, Department of Rare Disorders, Oslo University Hospital, Oslo, Norway; 4 KG Jebsen Centre for Neurodevelopmental Disorders, University of Oslo, Oslo, Norway; 5 Norwegian Centre for Mental Disorders Research (NORMENT), Division for Mental Health and Addiction, University of Oslo and Oslo University Hospital, Oslo, Norway

**Keywords:** Genetics, Diagnostic markers

**Fig. 1:**
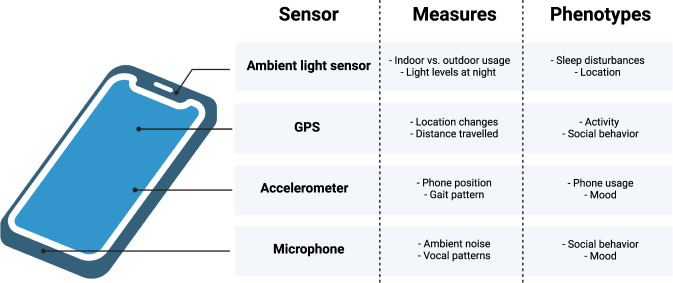
Smartphones. Modern smartphones contain various sensors that can be used to passively collect rich behavioral data that can complement traditional lab-based data types collected in molecular psychiatry research.

Recent years have seen a sharp rise in studies linking digital phenotypes to psychological functions [1]. Digital phenotyping has been defined as the prediction of psychological traits and states from digital variables, which typically includes mobile sensing via smartphone data logs (e.g., app use, call behavior), smartphone sensors (Fig. 1), and social media activity. The psychological sciences have been early adopters of digital phenotyping, however, there has been comparatively little progress in molecular psychiatry research. This is a missed opportunity to collect rich behavioral data that can help unravel the neurobiological mechanisms underlying psychiatric illnesses. The use of digital phenotypes can also be extended from its traditional applications to better understand the signaling systems underlying cognition and behavior. Here, we will demonstrate the potential of digital phenotyping in molecular psychiatry by illustrating possible applications for oxytocin research, which is a popular line of research in the field that can especially benefit from the multimodal nature of digital phenotyping.

Oxytocin is a nonapeptide that influences a diverse range of cognitive, behavioral, and somatic processes. Quintana & Guastella [2] proposed the Allostatic Theory of oxytocin to account for these diverse effects of oxytocin across a wide range of contexts, whereby the effects of oxytocin can be best understood by facilitating stability in changing environments. In other words, oxytocin helps enable behavioral and cognitive flexibility to promote organism stability (e.g., energy levels). While this theory was derived using a common approach in ethology (i.e., Niko Tinbergen’s “four questions”), this is a relatively novel concept for the biobehavioral sciences. Currently, this theory is mostly grounded on observations from animal research and comparative analyses between species. Although results from recent human research have been consistent with this theory (e.g., [3]), additional human studies across a range of contexts are required to provide more robust support. Digital phenotyping can facilitate the collection of an untapped source of behavioral data across a range of contexts to evaluate the Allostatic Theory of oxytocin and provide a better understanding of the oxytocin signaling system in general. The Allostatic Theory is well-suited to digital phenotyping given its emphasis on broad allostatic functions that benefit from the collection of multiple measures across domains.

A natural starting point in applying digital phenotyping principles to oxytocin research would be to investigate the influence of a course of intranasally administered oxytocin on behavior and cognition. For example, instead of relying on conventional self-reported retrospective reports of behavior that rely on memory, with existing smartphone technology, it is now possible to record behavior both passively and accurately in terms of how often an individual communicates with others (e.g., phone calls, text messages; see a series of call variables empirically investigated here [4]), as well as levels of physical activity [5]. Along with measuring the *frequency* of communication, digital phenotypes can also be used to evaluate the *content* of these communications (e.g., mood) and whether these communications are sent from the same or different locations. Smartphone app usage statistics can also be collected. The Allostatic Theory would predict that under conditions of uncertainty, oxytocin treatment would be associated with increased behavioral variability compared to placebo (e.g., locations, travel routes, app usage, communication patterns). Compared to measures collected in the laboratory, smartphone logs can provide more ecologically valid data regarding daily activities and responses to environmental variations.

**Fig. 2:**
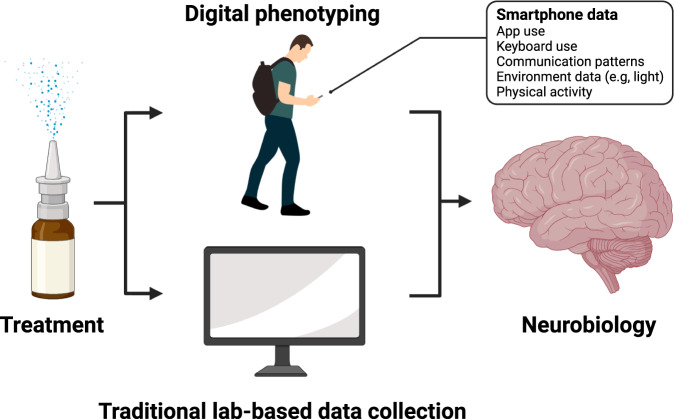
Digital phenotyping. Collecting digital phenotype data in molecular psychiatry research can help increase our understanding of the effectiveness of pharmaceuticals on neurobiology by complementing traditional lab-based measures of cognition and behavior with precise measures of behavior. For example, digital phenotyping can generate novel data on communication patterns, environmental conditions, and physical activity, which is not available with traditional lab-based data collection methods or spoiled by recall errors.

Another intriguing line of research is the linking of digital phenotypes and peripheral oxytocin concentrations via saliva across different contexts. It might be possible to uncover digital footprint patterns that are linked to varying oxytocin levels, which could function as digital biomarkers of peripheral oxytocin activity. Considering the Allostatic Theory of oxytocin, which emphasizes the energy regulation role of oxytocin [6], physical activity could also be recorded (e.g., distance walked per day, calories burned), which could reveal interesting relationships been oxytocin levels and energy expenditure. Researchers could also use smartphone data that can provide insights into variables, such as temperature [7], loudness of the surroundings [8], and body postures while using the smartphone via accelerometer data [9], to supplement oxytocin research. For example, with this kind of smartphone data, it is possible to detect late-night usage in bed while lying down, which could be used to index sleep disturbances. As the Allostatic Theory proposes that oxytocin might help humans to better adjust to environmental and energy regulation needs, relying more on environmental variables sensed from the smartphone will present researchers with an opportunity to better evaluate this proposal (Fig. 2).

Digital phenotyping can also be extended to investigating the role of genetics in psychiatric illness. We recently outlined how behavioral genetics and molecular genetic associations studies might benefit by not only relying on associations of genetic variables with self-reported questionnaire measures, but by also collecting behavioral measures via logged smartphone data [10]. Linking polygenic information related to variants in the entire oxytocin signaling pathway with over 150 genes to behavior may also open novel avenues to disentangle the complex role of oxytocin and its underlying mechanisms.

Of course, this approach can also be applied to other types of polygenic scores relevant to psychiatry research. Beyond intranasal oxytocin administration studies or linking oxytocin signaling pathway genetics to smartphone-log-data, such research designs could be used to investigate the effect of other psychopharmaceuticals such as SSRIs or ketamine/esketamine, and the signaling systems they target, on symptoms associated with major depressive disorder. For instance, measures of social withdrawal (e.g., more time at home or less contact with others) can be collected using smartphone data. Textual analyses of messages could also provide insights into negative emotionality (e.g., negative words or emoji). Research linking oxytocin-relevant brain processes to smartphone data is generally scarce at the moment. However, a recent study demonstrated a link between social app use and the dopamine system (via PET) [11], which is thought to operate very closely with the oxytocin system [3], thus providing some tentative indirect evidence for oxytocin’s role in social app usage.

While there is a growing ecosystem of tools and apps that support digital phenotyping, the study of logged smartphone and social media data has only very seldom been included due to its historical inaccessibility for most scientists in the field. To address this, we [4] and others (for an overview see [12]) have more recently introduced apps that can provide psychologists and psychiatrists custom-made tools for measuring diverse variables from the smartphone of the participants that can be combined with ecological momentary assessment, which only requires basic programming skills. Researchers can decide which variables to passively measure, such as call behavior and the frequency of app usage. In addition, researchers can prompt study participants to answer questions regarding their mental states on a daily (or other) frequency. Combining active and passive data can provide a deeper understanding of participant’s behaviors and thoughts, and these data layers can be added to available molecular data (e.g., genetics, brain imaging).

Despite the promise of digital phenotyping, the development of apps running on both iOS and Android operating systems can represent an obstacle, because comprehensive digital phenotyping is mostly still only possible on Android operating systems, as the iOS operating system offers less flexibility for system-wide data collection. The possibility of software updates disabling app features and designing the app to properly function across a wide range of smartphone models represent additional challenges for collecting digital phenotype data. Another problem touches upon psychometrics: for instance, how long needs a certain variable be tracked to get reliable and valid insights into the digital phenotype of interest?

One of the premier advantages of digital phenotype data is that it offers precise and ecologically accurate behavioral data compared to self-reported behavior, which is often inaccurate [13]. Prior to the advent of smartphone data collection, collecting ecologically accurate behavioral data required considerable resources (e.g., observational studies with at least two observers to calculate important interrater reliabilities). Another related benefit is that digital phenotyping provides the valuable opportunity to explore links between social media usage and psychiatric illness across the lifespan. Of note, lifespan investigations might still be hampered by lower usage levels of smartphones in older generations, although empirical work demonstrates that smartphone studies in older individuals may still be feasible [14]. This said, psychiatric illnesses are characterized by distinct developmental trajectories with adolescents exhibiting specific developmental windows of sensitivity to social media usage [15]. Early reports of broad deleterious effects of digital media use may have been overstated due to the inaccuracy of self-reported data [13], which highlights one benefit of passive smartphone data collection. The passive collection of smartphone data also reduces participant time burden, which can increase study recruitment and reduce dropout, and increase sample sizes due to the ubiquity of smartphones.

Digital phenotyping can provide many benefits, but it is critical to keep in mind that the richness of this data also introduces privacy and ethical challenges that need to be deftly navigated for the public to maintain trust in digital phenotyping research [16]. It is a challenge to safely record this sensitive data, analyze it properly in the context of the scientific question, and to ensure that data patterns cannot be used to re-identify persons. Moreover, digital phenotype data is still prone to biases that exist with other kinds of data collection that can influence data interpretation (e.g., socio-economic status) [17]. When keeping these potential pitfalls in mind, digital phenotyping can offer an unparalleled level of detail into the life of individuals that can broaden our understanding of psychiatric illnesses and help evaluate the effects of pharmaceuticals.
